# Clinical Records

**Published:** 1859-10

**Authors:** 


### CLINICAL RECORDS.

(From the *London Lancet.*)

*Tonics and Iron in Erysipelas of the Face and Scalp.*—We very frequently see the value of the treatment of erysipelas of the scalp and face by the exhibition of the muriated tincture of iron conjoined with tonics, and dusting the inflamed skin with flour, not neglecting proper attention to the chylopoietic viscera. We might refer to several recent instances in which the efficacy of iron has been marked, but shall content ourselves with noticing that of a woman in Guy's Hospital under the care of Dr. Wilks. She is fifty-seven years of age, and was admitted on the 6th inst., but the erysipelatous inflammation had set in a few days before that period, and extended all over the face and the scalp. Its intensity was not so great as to cause closure of the eyes, nor were the features altogether obliterated. The scalp was remarkably swollen, puffy, and extremely tender. When placed in bed, on her admission, the inflammation had apparently subsided, and evidences of desquamation were already manifest. Nevertheless, she was in a precarious condition, being very weak and low, and evidently requiring generous and supporting treatment. Twenty minims of the muriated tincture of iron were ordered every four hours, with eight ounces of wine, porter, and light nourishment. She now began to improve, and when we last saw her (on the 12th instant) she was sitting up in bed, with still some disfigurement of the features, and puffiness and usual tenderness of the scalp. Her improvement was uninterruptedly steady under the use of the steel, and she is making a good recovery. We have seen cases thus treated from the beginning with equal advantage.

*Excision of the Knee-Joint for Old Standing Disease.*—On the 7th instant we were present, at the Great Northern Hospital, when the knee-joint of a man, twenty-six years of age, was removed by Mr. Price. It was one of those cases of disease which he believes to be well adapted for the operation. It was surmised that the mischief was confined to the synovial membrane and the cartilages of articulation. On opening the joint purulent fluid escaped. The ends of the articulating bones were found in the condition expected: the synovial tissue had almost disappeared; the cartilages were entirely removed, except a few spots; while the exposed bone was healthy in appearance, vascular, but not ulcerated to any great extent. The patella was deprived of its cartilage, and was removed. We noticed that the operator, in opening the articulation, first reflected only on the skin and sufficient of its cellular connections, so that the infiltrated fat and loose tissues which generally abound about the joint when it has been long diseased, formed no part of the flap. Should any unhealthy inflammatory action set in, this altered structure is liable to slough and greatly complicate the treatment of the wound. The hæmorrhage was more copious than usual, the soft parts and periosteum being extra vascular. The limb was adjusted in a manner recommended by the operator, and up to the present time, the patient has expressed himself greatly benefited by the operation, his appetite and sleep having returned.

*Disease of the Sternum simulating Aneurism.*—The following case is one of great interest, and is still under treatment at the Hospital for Consumption, Brompton, under the care of Dr. Edward Smith.

An athletic man, aged thirty-two, engaged in a gunpowder factory, had felt palpitation of the heart, after moderate exertion, for twenty years. Fourteen years ago he had rheumatic fever during six weeks. He has at various times been much alarmed by explosions. He has been accustomed to make great muscular efforts, particularly in turning a crank or a mill, in which he had to use great effort in dragging toward himself. Whilst engaged in this violent labor about sixteen months ago he felt a sudden giving-way within the chest, and soon afterwards first perceived a bulging at the middle of the sternum.

July 7th, 1858. There is now a bony projection, beginning about two inches from the top of the sternum, extending downwards four inches, and transversely three inches, having its highest part opposite the third rib. There is no tenderness on pressure, but the surface is red and covered with hair. He has scarcely any internal pain, but there is a sense of stretching about the sternum, and at night he feels a little throbbing chiefly on the left side. There is no purr, nor any pulsation perceptible to the touch. There is a musical blowing with the second sound over and to the left of the sternum, and a non-musical and soft murmur about the apex of the heart. The bruit is not loud anywhere, but it extends to the top of the sternum and to a wide extent below. There is a rough systolic and a feeble diastolic bruit at the apex, and there is pulsation ardent and natural at the apex of the heart and its vicinity. The pulse is 76, full, even, and regular in both wrists when sitting, and the respirations are 23 per minute. No unusual pulsation in the carotid or subclavian arteries, nor any turgidity of the veins.

The case was thus obscure, but it wore a serious aspect, and a fear was entertained less it should be proved to be one of aneurism of the ascending aorta.

Sept. 1st. Again examined, and presents the same symptoms.

15th. He has had a little pain in the right breast, and a sense of pressure on each side of the chest when lying down.

Oct. 6th. There is more pain, and it is of a darting character; the tumor is a little larger; there is no dysphagia; his appetite is not good; and a careful examination of the lungs shows that there is lessened vesicular action. There is still a blowing diastolic sound, and it is sharper on the right of the sternum; arterial pulsation still regular. He is beginning to stoop somewhat, and there is insufficient respiration.

20th. He has suffered somewhat more pain at night, but pain has never been a prominent symptom. There is now fluctuation perceptible at the lower part of the tumor and in the space on the left of the sternum, but there is no thrill nor pulsation there. He has experienced one or two attacks of shivering.

At this period the case became less obscure, for it was almost certain that the pulsation was due to the presence of a little fluid in the anterior mediastinum. The case was now examined by a number of Dr. Smith's medical friends, and the general opinion arrived at was, that it was not a case of aneurism.

Nov. 3. Still in the same state.

11th. Dr. Smith showed the case to Mr. Fergusson, the consulting surgeon to the hospital, who regarded it as one of disease of the bones of the sternum. The patient would not permit an exploring needle to be used, as his club surgeon had informed him that he was suffering from an aneurism.

24th. No change.

27th. A small bladder of the size of half a hazel-nut has formed where the fluctuation was perceptible, but no discharge has taken place.

Dec. 8th. The health has improved, and the bladder is a little shrunken at the top.

21st. On the 18th there was a very small quantity of clear fluid discharged, which formed a small crust; and the bladder is a little shrunken.

Jan. 5th, 1859. The bladder is slightly enlarged, but there is no change in the condition of the tumor.

May 20th. Still in the same state, and able to do light work.

This case is very interesting from its obscurity in its earlier stages, and shows well how guarded the practitioner should be in forming an opinion as to the nature of such diseases, and more particularly in expressing any opinion to the patient. Its march has been very slow, and unmarked by any prominent symptom, and seems to be very much independent of any control on the part of the physician or surgeon. Dr. Smith's aim in treatment was to prevent local irritation and to maintain and improve the general health, but particularly to remove the habit of feeble respiration and to cause the diminution of the vesicular murmur, which constitutes the first stage of phthisis, and tends so frequently to the deposition of tubercle. This has been in great part effected.

*Tobacco-Pipe Stem in the Throat.*—The recent wound of the throat by a tobacco pipe, in which the carotid artery was tied by Mr. Ure, at St. Mary's Hospital, will be in the recollection of our readers. The ligature came away on the eighteenth day, and the poor man was progressing very favorably, but with the inconvenience of almost complete closure of the mouth, which had remained since the day of the accident. He was put upon a grain of sulphate of iron three times a day, with evident advantage. On the 22nd of June, he felt something in his mouth, and on introducing his two fingers withdrew the stem of the tobacco-pipe from beneath the left side of the tongue, where it had remained unsuspected and unobserved for several weeks. It measured two inches and three-quarters in length. The removal of this body permitted the mouth to open wider, and the rigidity of the muscles of the jaw to relax. No bad consequences have ensued, and as we had already predicted, a good recovery has taken place.

*Encephaloid Disease of the Epididymis.*—A lad sixteen years of age was admitted, on the 22nd ult., into University College Hospital, with encephaloid disease of his left testicle, which had grown within seven months to the size of a cocoa-nut. By the end of the next few days, it had increased nearly three inches, so no time was to be lost in its removal, which was performed by Mr. Erichsen on the 27th. The anterior part of the scrotum was red; the tumor was soft in front, but indurated posteriorly; and although the disease was extensive, the spermatic cord was unaffected. A section of the tumor showed the body of the testicle to be quite healthy, situated in the centre of the diseased mass which had originated in the epididymis. The wound was attacked with erysipelas the next day, which is prevalent just now, and temporarily retarded the healing action, but the boy is otherwise doing well.

We were present at the Middlesex Hospital on the 25th of May, when the right testicle was removed from an elderly man for the same disease. It originated in a blow ten months before, and had latterly much increased in size, until it was as large as a fœtal head. For three months after the blow no great inconvenience was experienced. From the general appearance of the man, there was no doubt that he had serious internal organic disease, which would endanger his life at a later period. He has recovered from the effects of the operation. A section of the tumor showed it to be the well-known form of the disease, with the development of several small cysts. Metallic sutures were used to bring the edges of the wound together.

*Nasal Carcinoma.*—We were lately shown a patient under Mr. Coulson's care at St. Mary's Hospital, who had a carcinomatous tumor in rather an unusual situation. It occupied the left side of the nose, was oval in shape, of the size of an almond, and was partly hollowed out by ulceration. He was admitted on the 24th of June, and stated that the disease commenced about a year ago, in the form of a small pimple over the left nasal bone, which slowly increased in size, became inflamed, and then ulcerated. Various caustics were employed—amongst others, strong nitric acid—for destroying the surface, followed by the application of the concentrated chloride of zinc. It is quite possible, with perseverance and attention on the house surgeon or dresser of the patient, in applying the caustics, that the ulcer may be got to heal. When we last saw it, it had an angry and irritable look, which had been somewhat increased during the prevalence of the great heat of the last few days.

*Useful Plan of Supporting Stumps after Amputation.*—At Guy's Hospital, for the last two years, Mr. Hilton has been in the habit of supporting the stumps of amputated thighs in a manner which is worthy of notice, from its cleanliness and convenience, together with the comfort accruing to the patient. It consists in applying a short and broad splint under the stump, which is elevated at an angle of forty degrees; beneath the splint is a small cushion, and a light bandage is applied over all. This permits of examination and dressing without the slightest disturbance to the patient, the stump always looks clean and healthy. The cases in which it is at the present moment employed are the following:

A young man, twenty-two years of age, was admitted on the 23rd March for extensive pulpy degeneration of the synovial membrane of the left knee, with incipient disease of the lungs. The former had existed for twelve months, and was making rapid inroads upon his health. The thigh was removed at its upper third on the 23rd ultimo; and when we examined the stump on the 5th instant, it had almost entirely healed, and looked remarkably clean and healthy from the way in which it was put up. The phthisical symptoms have completely subsided.

A second case was that of a man, aged forty-eight years, who, as we gather from the notes of Mr. Tuck, his dresser, was kicked by a horse on the knee twenty-one years ago, causing at that time a wound over the patella. He has been subject to frequent attacks of pain and swelling ever since. Three years ago the symptoms generally increased. Seven weeks back an abscess was opened at the side of the knee, and subsequently two openings had to be made to let out pus from the joint. The bones were much diseased, and he had suffered most acute pain. Considering his age and other circumstances, Mr. Hilton thought the most prudent course was amputation through the thigh, which he performed on the 5th instant, under chloroform. When placed in bed, the stump of this patient was carefully put up by Mr. Tuck in the manner already described, and we learn he is going on extremely well.

*Tumor of the Parotid.*—When a tumor extends somewhat deeply in the parotid space, its removal is often associated with a good deal of troublesome bleeding, even though no arterial trunk of any importance may be wounded. This fact we saw again verified, on the 15th of June, at University College Hospital, in a woman sixty years of age, who had been subject to a swelling in the left parotid space for from fourteen to sixteen years. Latterly, it had become active in its development, it was increasing in size, and getting soft at its most prominent part, where the integuments were discolored. This change Mr. Erichsen believed to be simple disorganization. The tumor was movable, and one portion dipped round the ramus of the jaw. Its attachments were considered not too deep for excision. It fully occupied the parotid region, although it did not involve the parotid gland; it was as large as the fist, and was in front and below the ear. It was successfully removed, together with a small portion of the temporal muscle, but the temporal artery was unavoidably divided in the course of the operation. This gave rise to considerable hæmorrhage, which was only controlled, after the lapse of some time, by the aid of many ligatures and the application of the perchloride of iron. The tumor proved to be fibro-plastic, undergoing degeneration, disintegration, and actual calcification in that part of it which was situated behind the ramus of the jaw. On the second day after the operation, she was attacked by erysipelas, and was in a precarious state for some days, but she is now slowly recovering, and the wound is fast closing.

Some weeks back, Mr. Quain removed a tumor from the neck of a woman aged about thirty-four, which had been growing for fourteen or fifteen years. She was pregnant at the time, but this did not prevent the wound from healing very rapidly. She was subsequently discharged from the hospital quite well.

*Double Fistula in Ano, treated by a Single Division of the Sphincter.*—Although at first sight it may seem to be a trifling matter, whether one or more divisions of the sphincter ani muscle be made in cases of complicated fistula about the anus, in reality considerable importance should be attached to it if the future comfort of the patient is considered. There can be no doubt whatever, as we heard Mr. Ferguson remark, at King's College Hospital, on the 2nd instant, that if there are two or more divisions of the sphincter muscle, subsequent union does not permit of such an amount of control over its functions as when one only is made. Being aware of the truth of this from experience, he treated the case of a young woman, who had what might be called a double fistula, in the following manner: Three years ago she had an abscess in the perinæum, which burst externally at the margin of the anus; probably a second formed, which also burst externally, but the two cavities merged into one. This aperture, on examination, was found not to communicate with the rectum, and was, therefore, what is called, in surgical language, *a blind external fistula*, with a double opening. Instead of running a bistoury through the sphincter in two places, as we have seen done by some surgeons, Mr. Ferguson divided the skin between the fistulæ, and laid open the cavity to which they were the outlets. He then cut through the sphincter nearest the upper fistulous opening, in the usual manner, and the wound was carefully dressed from the bottom. Thus, by a very simple proceeding, the case was converted into one of ordinary fistula in ano.

The practical surgeon will at once recognise the benefits to be derived from an avoidance of multiple divisions through the sphincter ani.

